# Past, Current, and Future Perspectives on Transplanting Acute Kidney Injury Kidneys

**DOI:** 10.3390/clinpract13040086

**Published:** 2023-08-14

**Authors:** Rachana Punukollu, Margaret Ryan, Suman Misra, Pooja Budhiraja, Stephanie Ohara, Kayla Kumm, Giselle Guerra, Kunam S. Reddy, Raymond Heilman, Caroline C. Jadlowiec

**Affiliations:** 1 Division of Transplant Surgery, Department of Surgery, Mayo Clinic, Phoenix, AZ 85054, USA; 2 Division of Anatomic Pathology, Mayo Clinic, Phoenix, AZ 85054, USA; 3 Division of Nephrology, Mayo Clinic, Phoenix, AZ 85054, USA; 4 Division of Surgery, Valleywise Health Medical Center, Creighton University, Phoenix, AZ 85008, USA; 5 Division of Nephrology, Miami Transplant Institute, Miami, FL 33136, USA

**Keywords:** acute kidney injury, kidney transplant, delayed graft function, organ shortage, donor pool

## Abstract

(1) Background: Acute kidney injury (AKI) kidneys have high non-utilization rates due to concerns regarding unfavorable outcomes. In this paper, we aimed to review the past, present, and future opinions on AKI kidneys. (2) Methods: A PubMed search was conducted for topics relevant to AKI kidney transplantation. (3) Results: Current short- and long-term data on AKI kidneys have demonstrated good outcomes including favorable graft function and survival. The role of procurement biopsies is controversial, but they have been shown to be beneficial in AKI kidneys by allowing clinicians to differentiate between reversible tubular injury and irreversible cortical necrosis. Machine perfusion has also been applied to AKI kidneys and has been shown to reduce delayed graft function (DGF). The incidence of DGF increases with AKI severity and its management can be challenging. Strategies employed to counteract this have included early initiation of dialysis after kidney transplantation, early targeting of adequate immunosuppression levels to minimize rejection risk, and establishment of outpatient dialysis. (4) Conclusions: Despite good outcomes, there continue to be barriers that impact AKI kidney utilization. Successful strategies have included use of procurement biopsies or machine perfusion and expectant management of DGF. With increasing experience, better use of AKI kidneys can result in additional opportunities to expand the donor pool.

## 1. Introduction

### 1.1. Historical Context

Acute kidney injury (AKI), an acute decline in renal function, is observed in upwards of 25% of deceased donors [1,2]. While AKI can be reversible in the non-transplant setting, repeated AKI events can increase the risk for chronic kidney disease [3,4]. In the transplant setting, deceased-donor kidney allografts with AKI are at a high risk of non-utilization and discard due to concerns related to organ quality, graft outcomes, and challenges with post-transplant management [5].

Early transplant data on AKI kidneys have suggested a higher risk for graft loss, delayed graft function (DGF), primary nonfunction, and lower estimated glomerular filtration rates (eGFR) compared to non-AKI kidneys [6]. Although these outcomes have been acceptable, they were still inferior to those of non-AKI kidneys, and there has been limited enthusiasm within the transplant community for broadening the use of AKI kidneys. However, as experience has grown, including more routine utilization of donation after circulatory death (DCD), newer data specific to AKI kidneys have begun to emerge showing that, for carefully selected donors with AKI, transplant outcomes were equivalent to non-AKI donors [7].

The use of preimplantation (procurement) biopsies is commonly described in current AKI data experiences. Although the general use of procurement biopsies is controversial and debated, the science supporting procurement biopsy use for AKI donors has established criteria guiding safe utilization [7,8,9]. Use of procurement biopsies in AKI donors allows for the assessment of irreversible cortical necrosis that can occur in severe AKI events. Procurement biopsies also allow transplant centers to screen for significant chronic changes that may have preceded AKI events. The inferior outcomes observed in earlier AKI studies may have occurred due to a lack of this biopsy information and the inability to fully assess all competing donor risk factors including AKI and chronic kidney disease. Since many studies have now demonstrated good outcomes with AKI donors, there have been some improvements in utilization [7,8,9,10,11]. Despite growing evidence of good outcomes with AKI donors, kidneys from AKI donors are still at a substantial risk for both non-procurement as well as non-utilization [12].

### 1.2. Ongoing Underutilization of AKI Kidneys

It is well known that the demand for kidney transplantation far exceeds the number of available donor kidneys. Between 2015 and 2017, only 39% of the patients on waitlists received transplants. For the remaining individuals, 20% of the patients were removed due to various reasons such as being too ill, 6% of the patients died, and 35% of the patients are still waiting for a transplant 3 years after being listed [5]. Given this knowledge, the transplant community has focused its efforts on improving utilization of all available donor organs. With respect to AKI kidneys, the problem begins even prior to organ recovery. A recent study that looked at United States data from 2000 to 2018 found that kidneys with a terminal creatinine > 2.00 mg/dL were 22 times more likely to not be procured compared to kidneys coming from donors with a terminal creatinine < 1.00 mg/dL [12]. Within the USA, the majority of kidney allografts come from deceased donors, yet the yearly non-utilization rate for deceased-donor kidneys has continued to rise [5]. According to the 2020 Annual Data Report regarding kidneys, the discard rate for deceased-donor kidneys reached its highest rate at 21.3%. Kidneys from older donors, donors with high kidney donor profile index (KDPI) values (>85%), donors with AKI, as well as donors with diabetes and a high body mass index are at the highest risk of non-utilization. Other reasons cited for discarding kidneys include abnormal biopsy findings, prolonged cold ischemic time (CIT), and anatomic issues [5]. The risk for non-utilization further increases when competing risk variables overlap, such as an AKI kidney coming from a high-KDPI donor with longer CIT.

As a result, there have been many efforts by the transplant community to identify reasons for discard and to improve utilization [13]. The role of procurement biopsies in facilitating kidney transplantation is controversial; many studies have shown that procurement biopsies may lead to erroneous information that does not correlate with clinical outcomes but rather facilitates discard opportunities [14]. It is, however, important to note that the role of procurement biopsies in assessing AKI donors is likely different and that the many transplant centers with expertise in these kidneys rely on procurement biopsies to guide clinical decision making and safety [7,8,9,15]. In response to these concerns, the Organ Procurement and Transplantation Network put forth a policy, in 2022, to establish minimal kidney donor criteria for a procurement biopsy [16]. As currently written, most AKI donors should meet the criteria for a biopsy within this policy (Table 1) [16]. Despite this policy update, the ability to reliably use biopsies for AKI kidneys is suboptimal. Procurement biopsies often continue to be read at hospitals unfamiliar with renal pathology and kidney transplantation. These biopsies are typically frozen section wedge biopsies that can be of poor preparatory quality, which makes interpretation by transplant centers difficult. The availability of electronic biopsy reviews has allowed for some improvement in this process by allowing transplant centers to review these biopsies firsthand. Other barriers to broader use of AKI kidneys include delays in allocation placement, lack of timely transportation logistics, and CIT. Decisions to expedite the placement of AKI kidneys by organ procurement organizations have been subject to recent scrutiny with some transplant centers suggesting that this process further exacerbates inequities in transplantation [17]. As supported by discard data, many kidneys come from AKI donors and necessitate allocation exceptions until there is broader and more consistent utilization within the entire transplant community. Even with expedited allocation, AKI kidneys often arrive with significant CIT which has increasingly been considered to be a modifiable factor in outcomes [10].

### 1.3. Current AKI Kidney Practices

With the availability of long-term data on AKI kidney outcomes, there has been increasing consideration and use of these kidneys by more transplant centers [8]. Initial early data from single centers have demonstrated higher rates of DGF, but without adverse impacts on one-year eGFR, acute rejection events, or patient and graft survival [7]. Similar outcomes have now also been shown long term, beyond the first year of transplant, both with single and multicenter data [8,10,18]. As experience has grown with AKI donors, transplant centers have been able to further expand the AKI criteria to other donor types including donation after circulatory death donors and donors with severe AKI on renal replacement therapy [9,19]. With this expansion, the data have likewise shown that the long-term outcomes of these carefully selected AKI kidneys are, in fact, similar to organs from equivalent KDPI donors without AKI [7,9,19]. In contrast to earlier reports, current data have shown that, although the incidence of DGF is significantly higher, the rates of primary nonfunction and the development of interstitial fibrosis have not proven to be statistically different [20]. In addition, AKI kidneys from DCD donors have also been shown to have equivalent outcomes to AKI kidneys from brain-dead donors, with equivalent one-year eGFR and graft survival [9]. Data on outcomes for high-KDPI donors with AKI are limited, but there is some evidence to suggest that high-KDPI kidneys (>85%) with severe AKI may have higher rates of primary nonfunction [15]. For high-KDPI donors with AKI, careful review of the procurement biopsy to exclude underlying significant chronic changes is very important to reliably predict post-transplant outcomes and to minimize risk for primary nonfunction. Although high-KDPI AKI kidneys can be transplanted successfully with acceptable long-term outcomes, they warrant careful consideration prior to utilization [15]. Thorough evaluation for underlying chronic changes, such as fibrosis, along with irreversible acute changes (cortical necrosis) are important in this context. At present, progression to fibrosis, either due to chronic or acute injury, is irreversible. The molecular mechanisms responsible for these histologic changes is fully understood; however, recent studies have focused on understanding these pathways. The identification of these mechanisms has the potential to yield therapeutic strategies in both chronic kidney disease and acute kidney injury which could be applied to further expand the use of AKI kidneys in transplantations [21,22].

**Figure 1:**
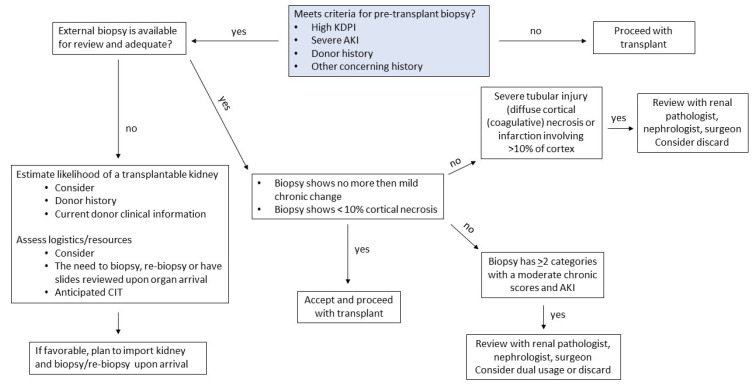
Mayo Clinic algorithm for assessing AKI kidneys with biopsy.

**Figure 2:**
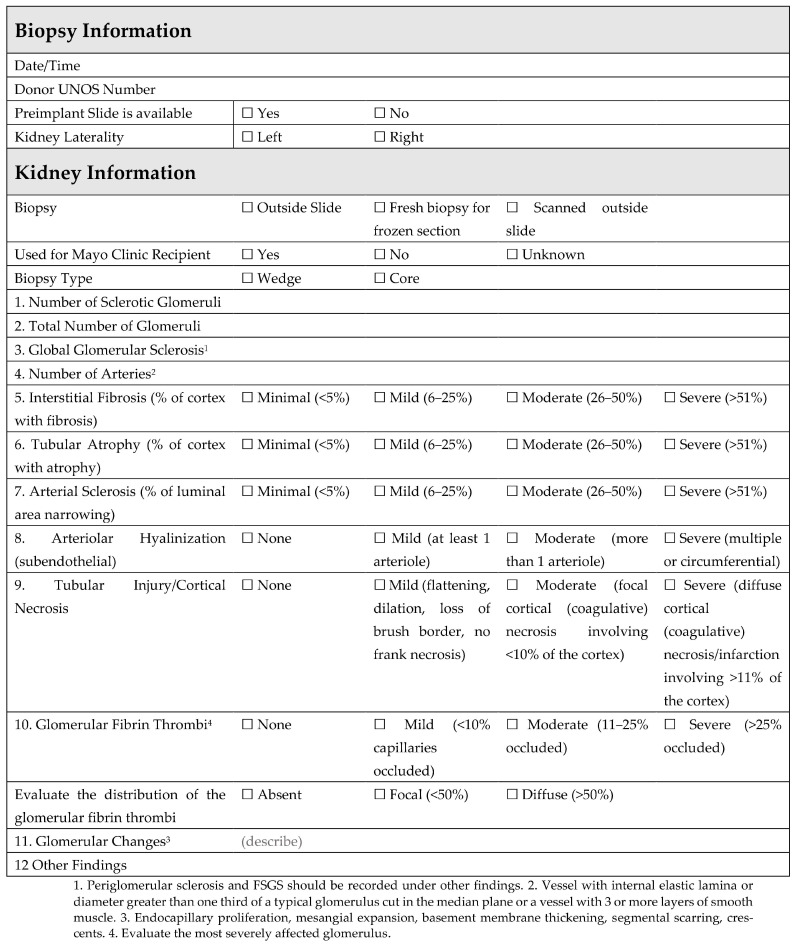
Mayo Clinic standardized form for a preimplantation biopsy review [31].

The success that our transplant center program has had in utilizing severe AKI kidneys is multifactorial, beginning with careful consideration of a donor’s clinical history and cause of death. The etiology of a donor’s AKI and an indication for renal replacement therapy is also pertinent. The duration of renal replacement therapy and anuria can provide insight into the severity of AKI. When possible, minimization of CIT and use of machine perfusion is ideal; however, this is not always modifiable due to late allocation offers and logistical complexity. Prior to proceeding with a transplantation, per our center’s protocol, all kidneys with AKI undergo a preimplantation procurement biopsy. All efforts are made to review these biopsies by our transplant center’s on-call pathologist, nephrologist, and transplant surgeon. Historically, this has largely involved physical review of the slide at the time of kidney allograft arrival. If the slide could not be provided by the organ procurement organization, then a second preimplantation biopsy was performed. These practices have been, at times, challenging with the addition of incremental CIT as well as unnecessary resource utilization if the biopsy read proved to be unsuitable for kidney transplantation (Figure 1). More recently, organ procurement organizations have made electronic biopsy reviews more readily available. When available, this option has allowed transplant centers to re-review a biopsy to ensure its suitable quality. The histologic parameters that have been taken into consideration in the decision to utilize the organ are shown in Figure 2. After kidney transplantation, familiarity with expectant management of DGF is also important. One of the most important aspects for the recipients is ensuring that they are aware of the likelihood of DGF, that this is a common and not worrisome event, and that there will likely be a need for some dialysis after kidney transplantation.

### 1.4. Role of Procurement Biopsies for AKI Kidneys

Reported data on kidney allograft biopsies, as a whole, have been heterogenous, in part, due to variability in reporting. Prior studies have shown that chronic changes in formalin-fixed paraffin-embedded postreperfusion biopsies correlate with inferior outcomes following deceased-donor kidney transplantations [23,24,25]. Among the studies that have not demonstrated an association with glomerulosclerosis, the data have largely been based on procurement wedge biopsies [26,27,28]. Details specific to biopsies, including technique and timing, have been vague in other studies that have not shown an association between glomerulosclerosis and outcomes [26,29]. Procurement biopsies in the United States are most commonly wedge biopsies that are processed as frozen sections due to time constraints, often using a single stain. These biopsies are often interchangeably referred to as preimplantation or procurement biopsies. There are advantages and disadvantages to both core needle and wedge biopsies. The quality of both biopsy types is dependent on the technique in which they are completed. Deeper wedge biopsies are often avoided at organ recoveries due to concerns for post-transplant complications. As a result, most wedge biopsies sample only the subcapsular region and may not accurately represent the true renal architecture [30]. Frozen section biopsies are also considered to be inferior in quality compared to formalin-fixed biopsies due to artifacts related to the preparation and thickness of the biopsy sections. In fact, good wedge biopsies not restricted to the subcapsular cortex can, however, be superior to needle biopsies [31]. In the “Banff histopathologic consensus criteria for preimplantation kidney biopsies” study, the authors Liapis et al. found better agreement among pathologists when reading wedge biopsies compared to reading needle biopsies [31]. In this context, it is important to anticipate what these limitations might be when interpreting biopsies and to adjust the interpretations accordingly. A strategy to counteract these limitations is to have the biopsies reviewed by an experienced renal pathology team that can comment on the quality and sample adequacy. The clinical interpretation of a biopsy is important, and a procurement biopsy should not be used for decline but rather to guide decision making on recipient selection as well as the potential need for dual versus single adult kidney transplantation.

The evaluation of a preimplantation biopsy by a pathologist is controversial. In some part, this is related to variability in performance and reporting. To improve the standardization of preimplantation kidney biopsy procurement, the Organ Procurement and Transplantation Network has proposed new guidelines, wherein a procurement biopsy is performed on a specific subset of organ donors [16]. With these new guidelines, among the additional criteria (Table 1), organ procurement organizations will be required to obtain a kidney biopsy on any deceased organ donor with urine output <100 mL in 24 h or having received hemodialysis during the current hospital admission. Procurement biopsies in the United States are most commonly wedge biopsies that are processed as frozen sections due to time constraints, often using a single stain. They are frequently read by an on-call pathologist who may not specialize in kidney pathology or kidney transplantation. Until recently, procurement biopsies were variably reported. To comply with the minimum donor criteria guidelines (Table 1), the Organ Procurement and Transplantation Network has also approved a policy to standardize kidney biopsy reporting and data collection. This policy requires that biopsy data are reported in a typed (not handwritten) report, using a standardized pathology report [32]. With these new requirements, less variability in kidney biopsy usage and reporting is anticipated.

The value of procurement biopsies continues to be controversial, and some organ procurement organizations have opposed implementation of the updated minimal donor criteria policy because they believe the additional requirements for kidney biopsies will adversely impact kidney utilization. We do not recommend routinely using a procurement biopsy in younger, low-risk donor kidneys. However, in our center’s experience with transplanting kidneys from AKI donors, we have found that a procurement biopsy can be valuable. Transplant centers must try to safely utilize all available organs, pair the correct organ with the correct recipient, and ensure that that they are attaining appropriate short- and long-term quality goals. In some circumstances, procurement biopsy data add valuable information to this decision-making process. By comparison, a primary goal for organ procurement organizations is to increase successful placement of donor organs. In this context, the metrics by which transplant centers and organ procurement organizations are held are not always congruent.

**Figure 3:**
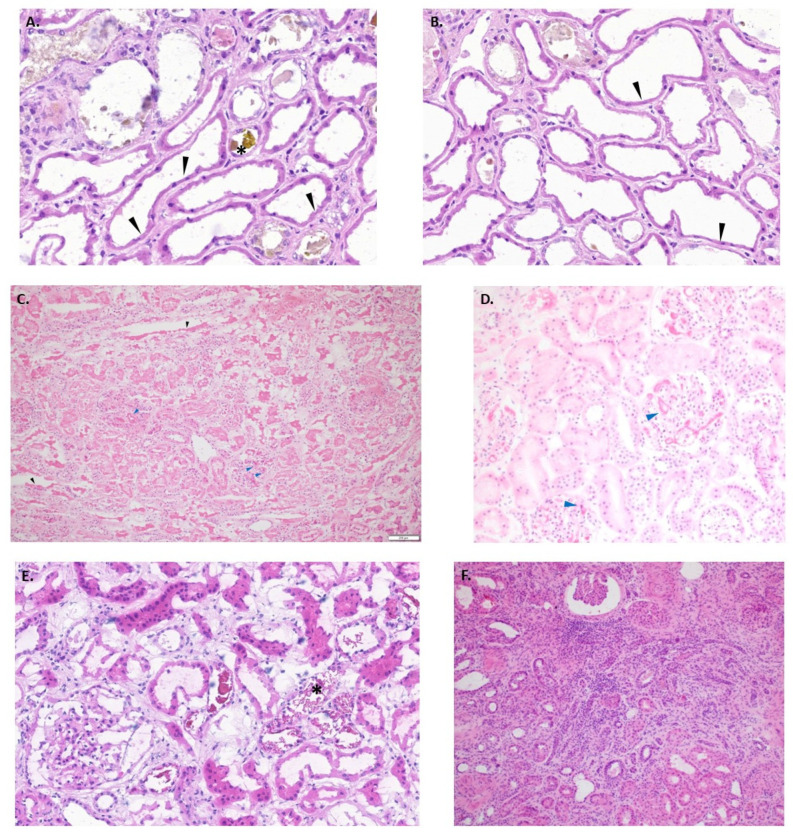
Kidney biopsy findings: (**A**) At 20× magnification, shows acute reversible tubular injury (black arrows) with bile casts (asterisk), flattening of tubular epithelium, dilation of tubular lumen, loss of brush border; (**B**) at 20× magnification, shows acute reversible tubular injury; (**C**) at 20× magnification, shows cortical necrosis (black arrows) with glomerular fibrin thrombi (blue arrows), see loss of all cells and nuclei lining the tubules (black arrows); (**D**) at 20× magnification, shows glomerular fibrin thrombi without associated cortical necrosis; (**E**) at 20× magnification, shows cast material consistent with hemoglobin or myoglobin (asterisk); (**F**) at 10× magnification, shows severe interstitial fibrosis and tubular atrophy.

At our institution, a standardized online fillable form is utilized within pathology for all requested preimplantation biopsies (Figure 2). The biopsy is reviewed, and histologic findings are documented, including all criteria set forth by the Banff histopathological consensus [31] for preimplantation renal biopsy. Preimplantation biopsies from AKI kidneys may show a multitude of histologic findings, including acute changes ranging from mild acute tubular injury (reversible) (Figure 3A,B) to frank cortical necrosis (irreversible) (Figure 3C). The extent of cortical necrosis should be assessed carefully as this is an irreversible process and these areas are replaced with tubular atrophy on subsequent biopsies. The presence (and extent) or absence of glomerular fibrin thrombi is also documented (Figure 3D). Glomerular fibrin thrombi are commonly observed in AKI donors, particularly if there is an associated head injury. AKI kidneys with focal glomerular fibrin thrombi and no cortical necrosis can be safely used for transplant [33]. In the setting of donor hemolysis or rhabdomyolysis, tubular hemoglobin or myoglobin cast material may be appreciated and should be documented (Figure 3E). Chronic changes such as glomerular sclerosis, interstitial fibrosis (ci), tubular atrophy (ct), fibro intimal thickening of arteries (cv), and arteriolar hyaline (ah) are also evaluated (Figure 3F).

Post-perfusion biopsies are used in concert with preimplantation biopsies and provide additional insight. The presence of similar pathologic findings in both pre and post implantation biopsies from a patient allow for the deduction that the process is donor derived, as opposed to de novo. Additionally post-perfusion biopsies of AKI kidneys allow pathologists to assess for any acute progression of tubular injury/necrosis and provide a well processed paraffin-embedded formalin-fixed piece of tissue for more complete histologic evaluation and for additional work-up, should this be necessary in the future.

### 1.5. Preservation Methods for AKI Kidneys: Static Cold Storage vs. Machine Perfusion

Static cold storage (SCS) is the most common method of preservation of organs due to its ease of availability and low cost. Marginal and high-risk kidneys are being increasingly accepted and used by transplant centers to meet the demands of ongoing organ shortage and recipients on waitlists. Such kidneys are more susceptible to ischemia-related reperfusion injury, along with higher rates of DGF and incremental risk for primary nonfunction [34]. Over the past two decades, new preservation techniques including ex situ machine perfusion have been shown to improve allograft function, reduce the incidence of ischemia reperfusion injury, and to improve drug delivery [35]. Although there are different types of machine perfusion based on the temperature, timing, and duration of the perfusion, hypothermic machine perfusion is the most widely used in kidney transplantation [34].

In an international randomized controlled trial that randomized one kidney to HMP and the other kidney to SCS, kidneys undergoing HMP had reduced risk and duration of DGF and higher one-year graft survival [36]. Other studies have shown similar results utilizing HMP, along with reduced economic burden due to reduced DGF rates [37,38,39] (Table 2). HMP potentially has the most benefit in kidney allografts with the most at-risk characteristics. In the MTI experience, use of HMP allows for increased utilization of kidneys with a higher KDPI and longer CIT [39]. Although there are no standard indications for HMP, it has shown clinical benefits in extended-criterion donor kidneys, DCD kidneys, and other marginal kidneys [39,40]. Recently, the Netherlands implemented HMP as their national standard preservation method to preserve deceased-donor kidneys and they have observed a significant reduction in DGF [41].

A single-center observational study at the Miami Transplant Institute (MTI) reported outcomes comparing the use of SCS-preserved kidneys with kidneys preserved with SCS at procurement and hypothermic machine perfusion (HMP) on arrival. The results showed an overall decrease in DGF, as well as slow graft function rates and low acute rejection rates compared to the use of only static cold storage [42]. In this study, the use of machine perfusion increased the mean CIT from 26 to 32 h and the mean KDPI from 49 to 61% [38]. In addition to decreasing the duration and incidence of DGF, HMP also allows for additional kidney allograft assessment based on perfusion parameters. The use of HMP to assess kidney allografts is a process consistently used by the MTI [43]. In the MTI experience, machine perfusion is used to optimize the kidney allograft and the greatest benefit is observed in those allografts with the most risk, including kidney allografts from AKI, DCD, and high-KDPI donors. Within this protocol, SCS is ideally limited to <24 h but with acceptable total ischemia times, including that of machine perfusion, exceeding 40 h. Machine perfusion parameters at the time of transplant generally include flows >100 mL and RI of <0.40 [43].

Since discard is often common with severe AKI kidneys due to concern for prolonged DGF, normothermic machine perfusion may help to decrease the DGF duration in AKI kidneys [44]. Normothermic machine perfusion has been recently gaining popularity in other areas of solid organ transplantation, namely lung, liver, and heart transplantations, and has begun to be increasingly used by a number of transplant centers around the world for kidney transplantation [45,46,47,48]. In contrast to HMP, the conditions are designed to mimic a more physiological environment at a near normal or subnormal body temperature. Different solutions are utilized in normothermic machine perfusion including red blood cell-based solutions, artificial hemoglobin solutions, whole blood, plasma, and acellular solutions [49].

Oxygen carriers are supplied with a crystalloid/colloid solution, oxygen, insulin, and various other nutrients to support metabolism [44,45,46]. Normothermic machine perfusion has been shown to be successful in utilizing organs such as the heart, lung, and liver from marginal donors by safe preservation, a reduction in ischemia-reperfusion injury, and DGF compared with conventional SCS, leading to successful organ transplantation [50,51,52,53]. However, the use of normothermic machine perfusion in clinical kidney transplantation is extremely limited [34], which is, in part, due to cost management and reimbursement [54]. The literature on outcomes using normothermic machine perfusion in AKI kidneys is almost nonexistent. A Cambridge study has shown the potential of NMP in decreasing DGF in marginal kidneys as compared with SCS [55]. According to a small preliminary study, normothermic machine perfusion AKI kidneys showed slightly improved eGFR at 3 and 12 months compared to its mate kidneys preserved using SCS, which was statistically insignificant [34]. However, a single-center study of five paired AKI kidneys compared normothermic machine perfusion to SCS and did not find a significant difference in the rate or duration of DGF despite a slight improvement in eGFR [34]. Larger multicentric randomized controlled trials need to be designed to address the reconditioning effect of normothermic machine perfusion in AKI kidneys.

### 1.6. Duration of DGF

AKI kidneys have a higher risk of DGF than non-AKI kidneys, and the severity of AKI further increases this risk [7,11]. A registry-based study in the USA found DGF rates of 25% for acute kidney injury network (AKIN) Stage 1, 32% for Stage 2, and 51% for Stage 3 [11]. The rate of DGF can be as high as 90% for donors with AKI needing temporary renal replacement therapy [19]. The duration of DGF also increases with the severity of AKI stage [34]. Additionally, other donor factors associated with increased DGF duration include longer cold and warm ischemia times, need for inotrope use in donors, older donor age, donor DCD status, and donor hypertension [11,56]. Historically, DGF has been reported to be a binary outcome. Although simplistic, this current definition likely limits a correlation with clinical outcomes. More granularity in the definition of DGF, including its duration and etiology, would likely help to better interpret clinical outcomes and guide management.

The data on the impact of DGF on graft function and rejection are conflicting, likely due to variations in study populations, the center practices of induction agents, and timings for initiating calcineurin inhibitors. In non-AKI kidneys, the studies in the United Kingdom and the Australian and New Zealand Dialysis and Transplant Registry have reported increased graft loss with longer DGF duration [57,58]. These studies have also reported a higher risk of acute rejection with DGF duration likely due to lower usage of depleting agents for induction [59].

In a single center study of 1018 patients with DGF, the use of AKI kidneys was not associated with poor graft survival or increased risk of fibrosis at the 4-month protocol biopsy compared to 696 allografts without DGF [56]. The graft function was predicted by KDPI rather than DGF duration. The use of severe AKI kidneys that often come from younger donors may explain the good graft function despite longer DGF duration; 53% of recipients received donor acute kidney injury network stage 2 or more kidneys in the DGF group versus 13.5% of the recipients in the non-DGF group. Furthermore, the risk of acute rejection was also not higher with DGF duration, likely due to early initiation of calcineurin inhibitors and the use of lymphocyte-depleting agents in recipients < 65 years. Other studies comparing AKI versus non-AKI donor kidneys have also reported no difference in acute rejection [7,59].

### 1.7. DGF Management

The management of DGF after kidney transplantation can be challenging and involves consideration of many factors including immunosuppression and volume status (Table 3). Immunosuppression management in the setting of DGF can be difficult and historical data have recommended early minimization of calcineurin inhibitors to potentially mitigate the duration or severity of DGF. A meta-analysis of 34 studies from 1988 to 2007 concluded that patients with DGF had a 49% pooled incidence of acute rejection compared to 35% in non-DGF patients [60]. The higher risk of acute rejection in these older studies may be related to delayed initiation of calcineurin inhibitors, thereby resulting in lower calcineurin inhibitor exposure in recipients with DGF. More recent studies have included routine utilization of induction therapy, including induction with lymphocyte depleting therapies which may have also resulted in lower rates of rejection [61]. A recent survey of centers across the USA and Canada suggested that transplanting kidneys with higher risk for DGF did not impact their decision of induction in the recipient [62].

The management of DGF after kidney transplantation is variable across transplant centers. A recent survey study of U.S. and Canadian transplant centers showed 20–40% DGF, with most centers reporting longer length of hospital stay [62]. Muth et al. showed that outpatient management of DGF with a dedicated DGF clinic was associated with shorter length of hospital stay and lower risk of acute rejection with no impact on patient or graft survival [63]. Kim et al. studied the financial impact of utilization of DGF which was associated with an approximately USD 18,000 increase in mean costs [64]. A study by our center showed that patients with DGF with early readmissions of two or more had worse graft outcomes. This subgroup was more likely to have diabetes and longer dialysis vintage time [65].

Transplant centers should develop a clinical protocol for managing patients who develop DGF. It is important to counsel patients on the likelihood of DGF based on risk factors. Careful assessments of urine output, volume status, and electrolytes are necessary early after transplantation to determine the need for initiation of dialysis. Anticipation of the need for vascular access for dialysis in DGF patients is important. Beta-blockers and clonidine should be restarted at a lower dose to prevent rebound phenomenon and avoiding ACE-inhibitors (ACE-i) and angiotensin receptor blockers (ARB) immediately after kidney transplantation is usually advisable. Early initiation of calcineurin inhibitors and maintaining an adequate calcineurin inhibitor trough level should decrease the risk of acute rejection. If available, the utilization of machine perfusion may be considered in donors with a longer CIT to decrease the risk of DGF [36].

At our center, we have adopted a clinical protocol for discharging patients with DGF on post-transplant Day 2 or Day 3. We follow these patients with outpatient assessments that include laboratory investigations three times a week and outpatient dialysis until DGF resolves. We start discharge planning on the day of transplant with the assistance of nursing staff and case management. The reduction in length of hospital stay translates to a cost reduction resulting from decreased utilization of hospital resources. Dialysis management should include careful assessment of post-transplant target weight based on clinical findings in order to avoid an abrupt drop in blood pressure. We advise that patients with DGF lasting more than 14 days should receive an ultrasound and biopsy of the allograft to rule out rejection or other unexpected complications.

## 2. Conclusions

AKI in donors is increasingly common, and despite good long-term outcomes, there continue to be significant barriers impacting discard and utilization. Careful screening of donors with AKI is important. Reviewing donor clinic history, procurement biopsies, and machine perfusion parameters have been strategies employed by transplant centers to avoid unfavorable outcomes. DGF frequently occurs with AKI kidneys and its incidence and duration increase with donor AKI severity. The success of normothermic machine perfusion in other solid organs presents an opportunity for more standardized access to machine perfusion for kidney transplantation. In addition to donor selection, post-transplant management plays an important role in successful outcomes for AKI kidney allografts. This includes patient counseling, early initiation of immunosuppression to reduce risk for rejection events, and frequent assessment of volume status. With increasing experience, there are additional opportunities to further expand the donor pool by using AKI kidneys. Future opportunities include ongoing standardization of procurement biopsy reporting, increasing availability of machine perfusion, and improving the definition and reporting of DGF.

## Figures and Tables

**Table 1:** The Organ Procurement and Transplantation Network’s minimum kidney donor criteria to require a biopsy.

Organ Procurement Organizations Must Make a Reasonable Effort to Ensure That a Procurement Kidney Biopsy Is Performed for All Donors Meeting Any of the Following Criteria, Excluding Donors Less than 18 Years Old:
- Anuria or a urine output of less than 100 mL in 24 h during the most recent hospital admission or in the course of donor management.
- Donor has received hemodialysis or other renal replacement therapy during the most recent hospital admission or in the course of donor management.
- History of diabetes, including hemoglobin A1c of 6.5 or greater during donor evaluation and management.
- KDPI that is greater than 85 percent.
- Donor age is >60 years or older. - Donor age is 50–59 and meets at least two of the following criteria: History of hypertension. Manner of death: cerebrovascular accident. Terminal creatinine > 1.5 mg/dL.

**Table 2:** Comparison of kidney transplantation outcomes using HMP compared to SCS.

Study	Study Design	Risk of DGF	Duration of DGF	PNF	Acute Rejection	Graft Survival	Economic Burden
Moers, 2009 [36]	Paired RCT	↓	↓	↔	↔	↑	-
Tingle, 2020 [37]	Meta-analysis of HMP vs. SCS RCT’s	↓	-	↔	-	↑	↓
Gasteiger, 2020 [38]	Retrospective propensity score-matched analysis	↓	-	-	-	↔	-
Brat, 2021 [41]	Prospective HMP arm, historical retrospective SCS arm	↓	↓	-	-	↔	-

↑, increase; ↔, no change; ↓, decrease; -, study did not investigate.

**Table 3:** DGF management recommendations.

DGF Management
**Inpatient** - Counsel patients on likelihood of DGF. - Initiate dialysis early if indicated. - Assess for the need of vascular access. - Restart low dose beta blockers and clonidine. - Avoid ACE-i/ARB if possible. - Start calcineurin inhibitors therapy early after transplant (post-transplant day 1–2). - Target adequate calcineurin inhibitors trough levels. - Engage care management team and nursing team early for discharge planning. - Early kidney transplant education for patient and caregiver. - Hospital discharge on post-operative day 2 or 3, if clinically stable.
**Outpatient** - Outpatient lab and provider visit 2–3 times per week until DGF resolves. - Dialysis at an outpatient facility 3 times a week. - Try to avoid hypotension during dialysis which may delay DGF recovery. - If DGF persists >14 days, proceed with an outpatient ultrasound and biopsy.

## Data Availability

Not applicable.
